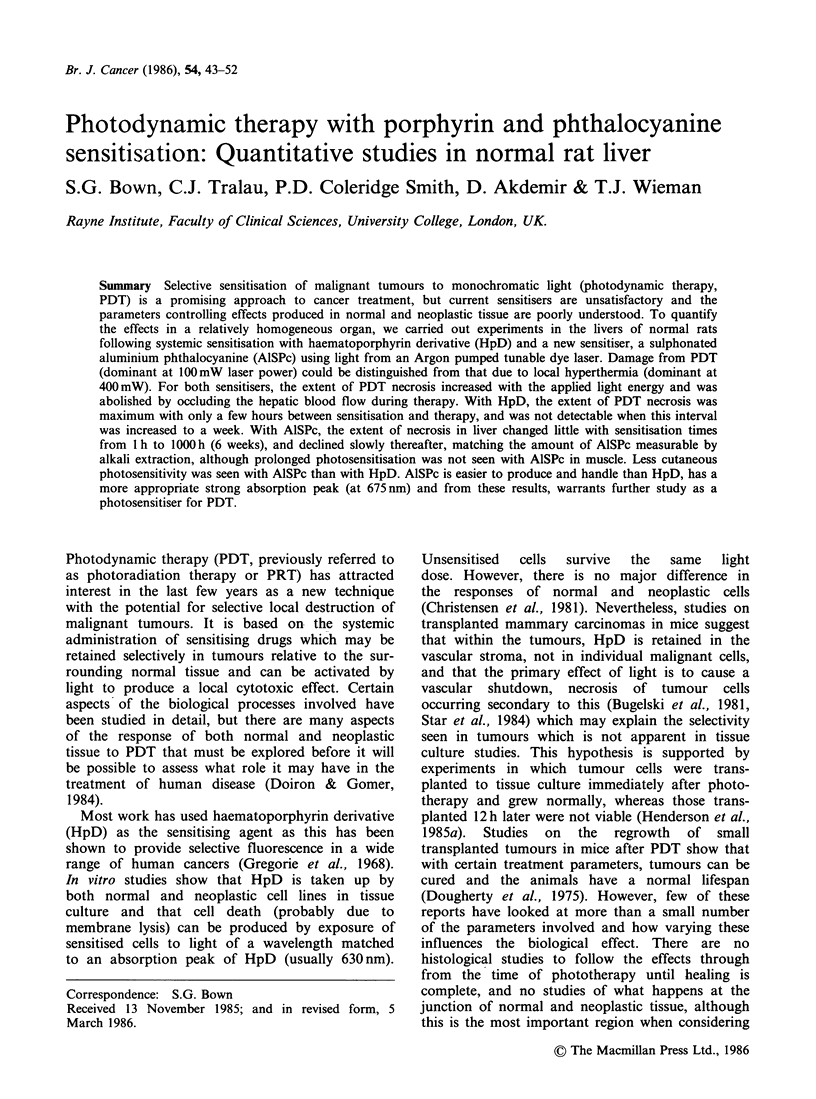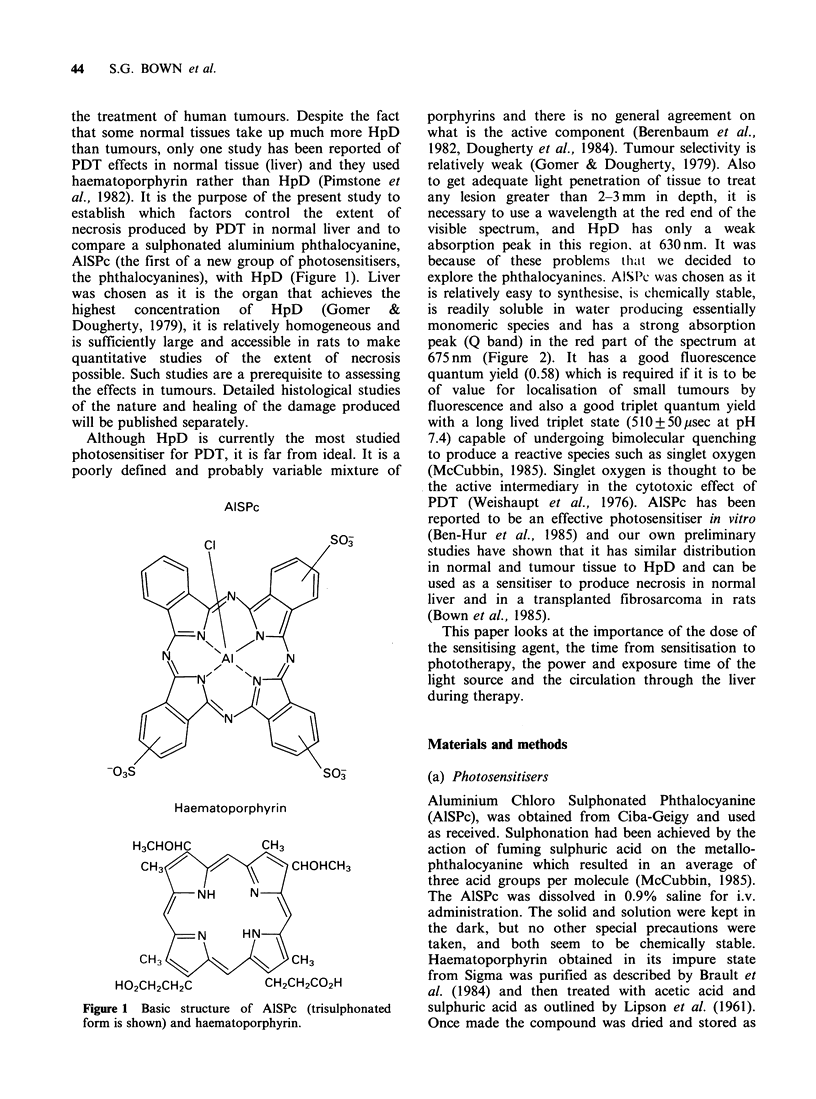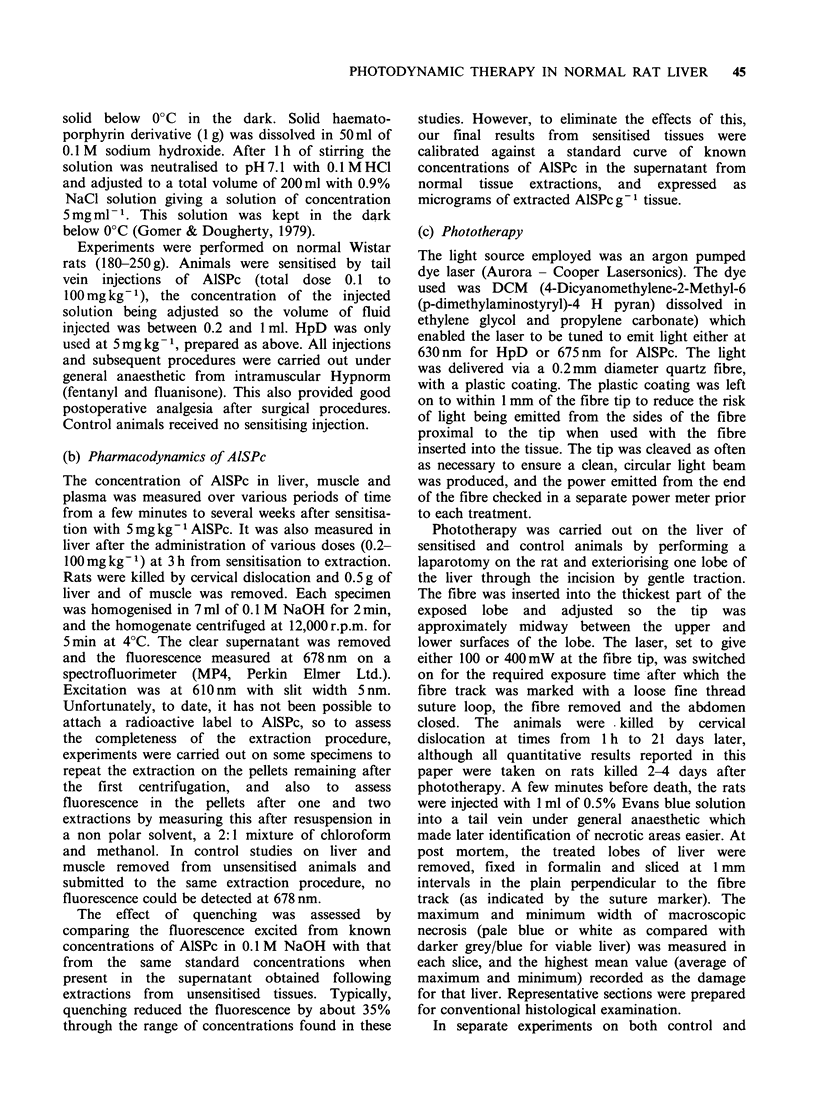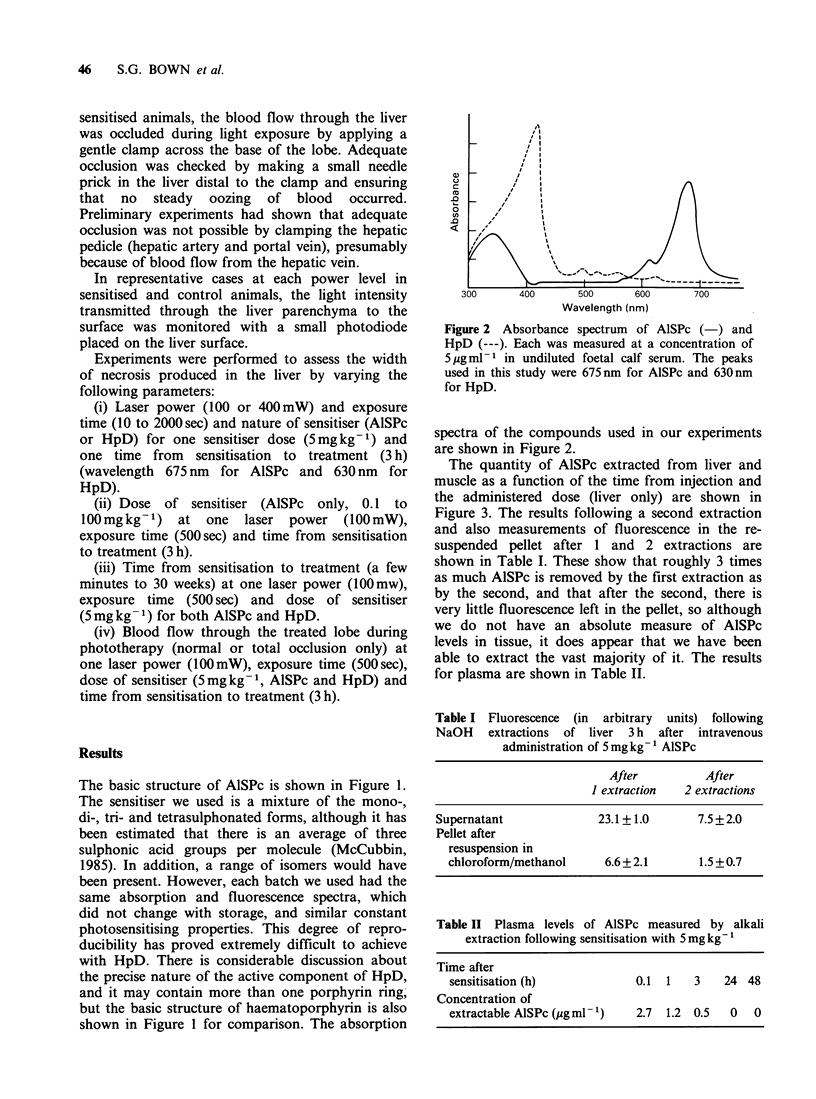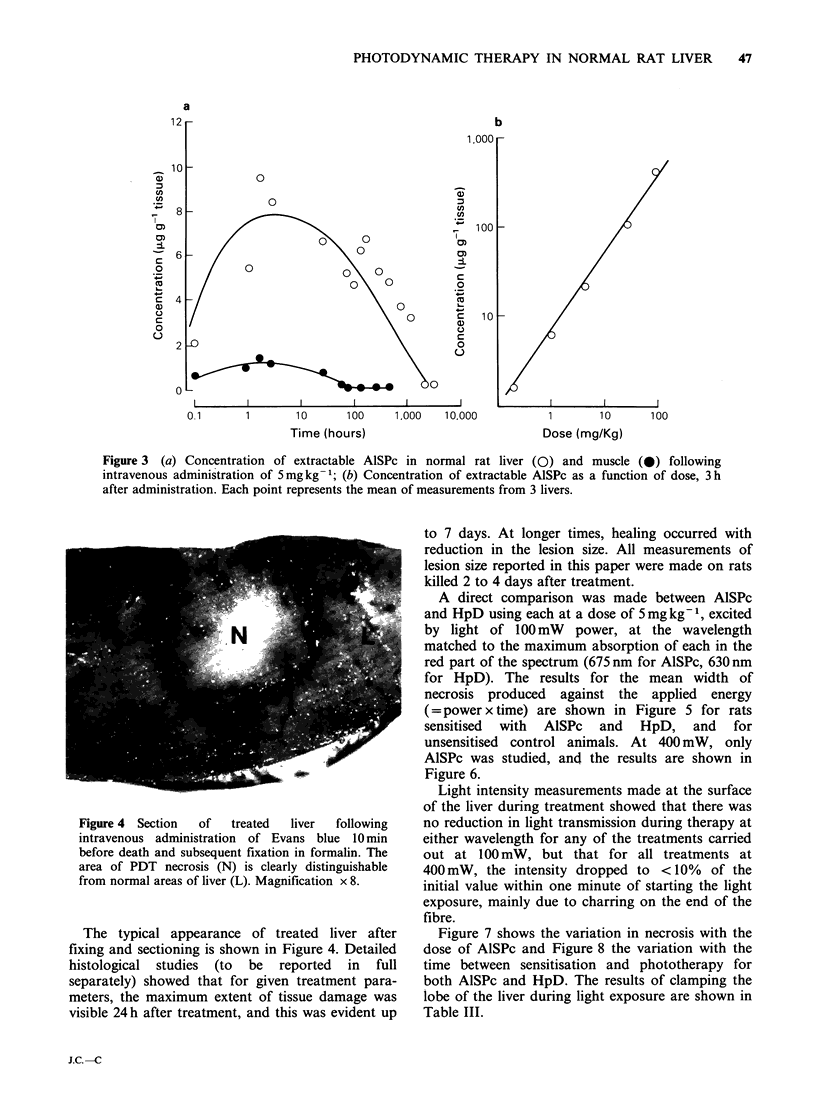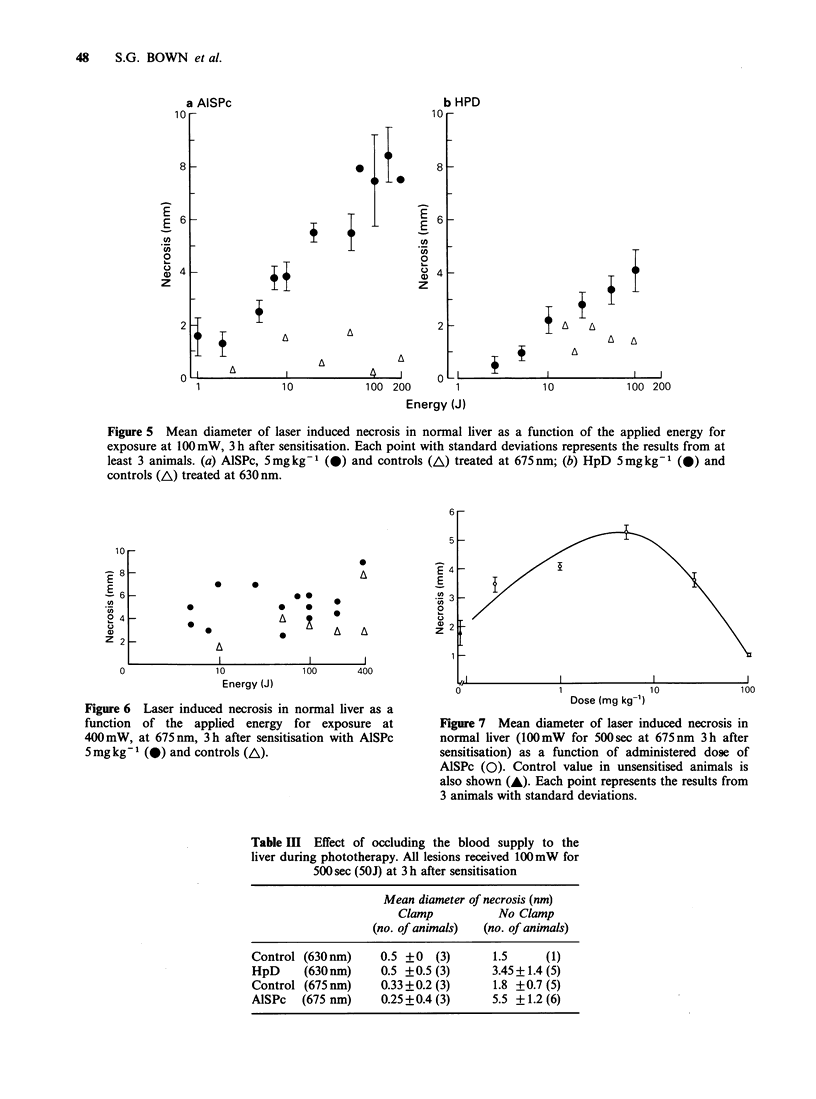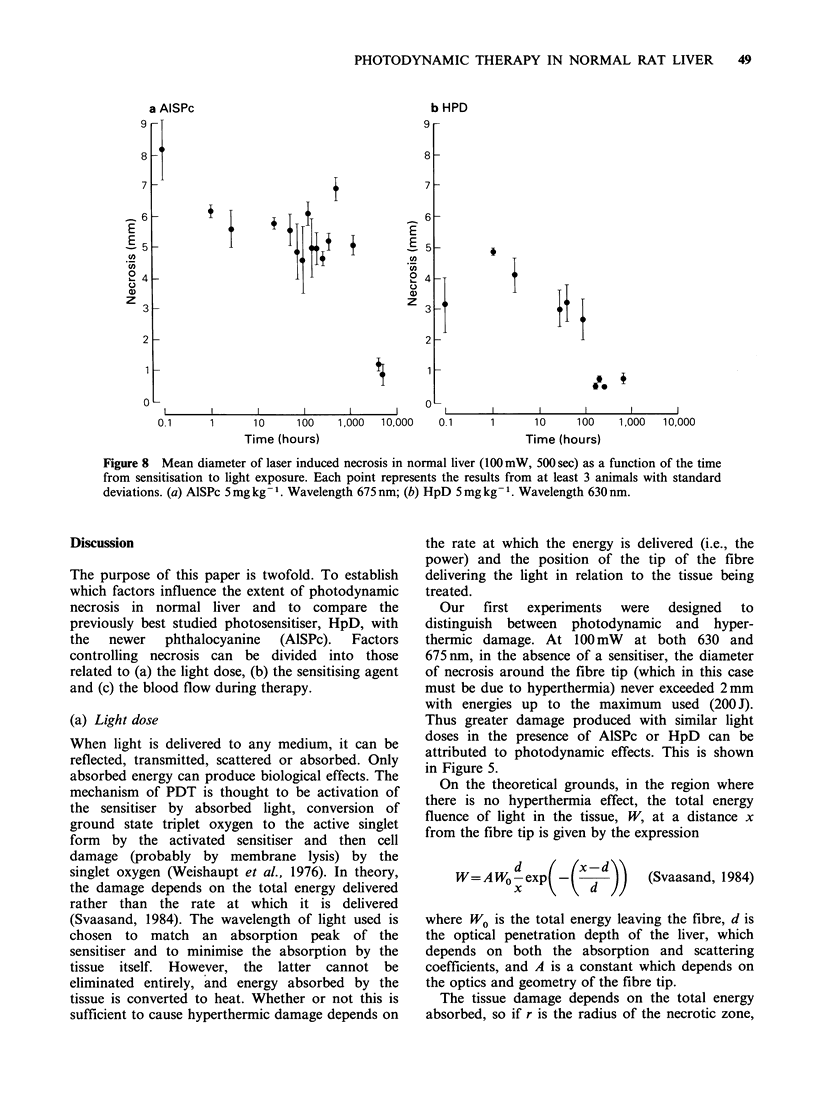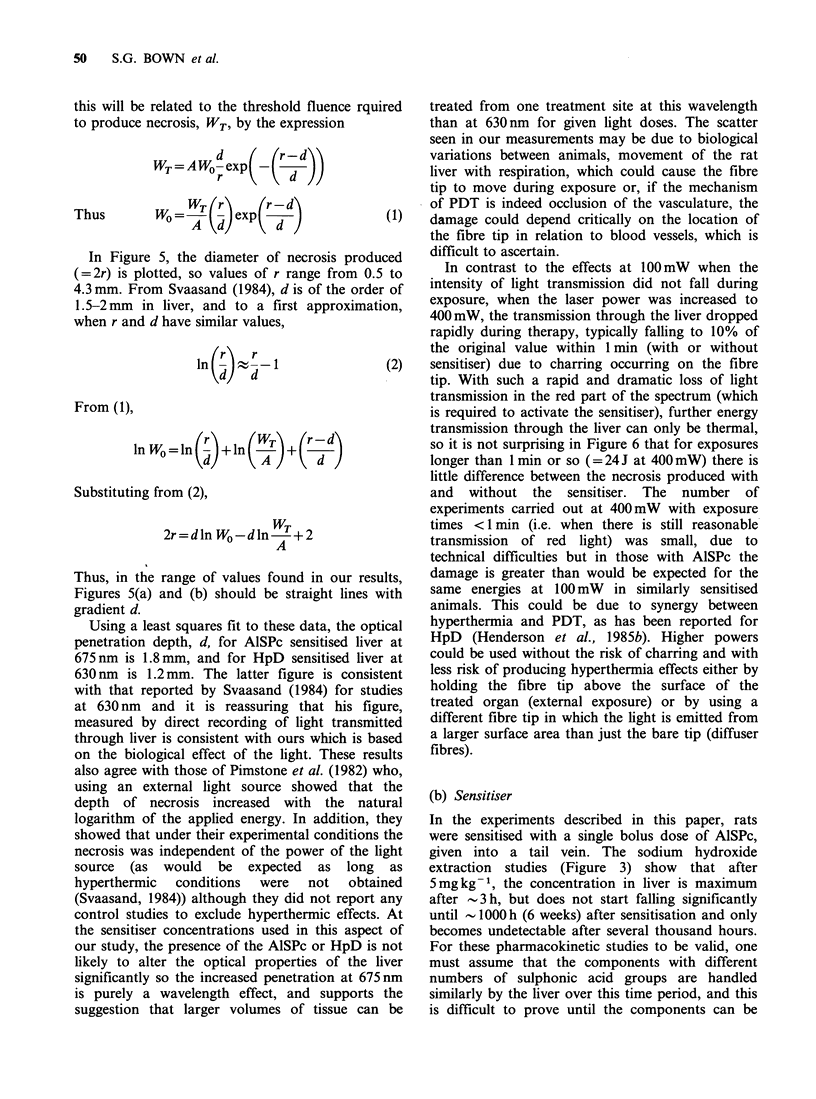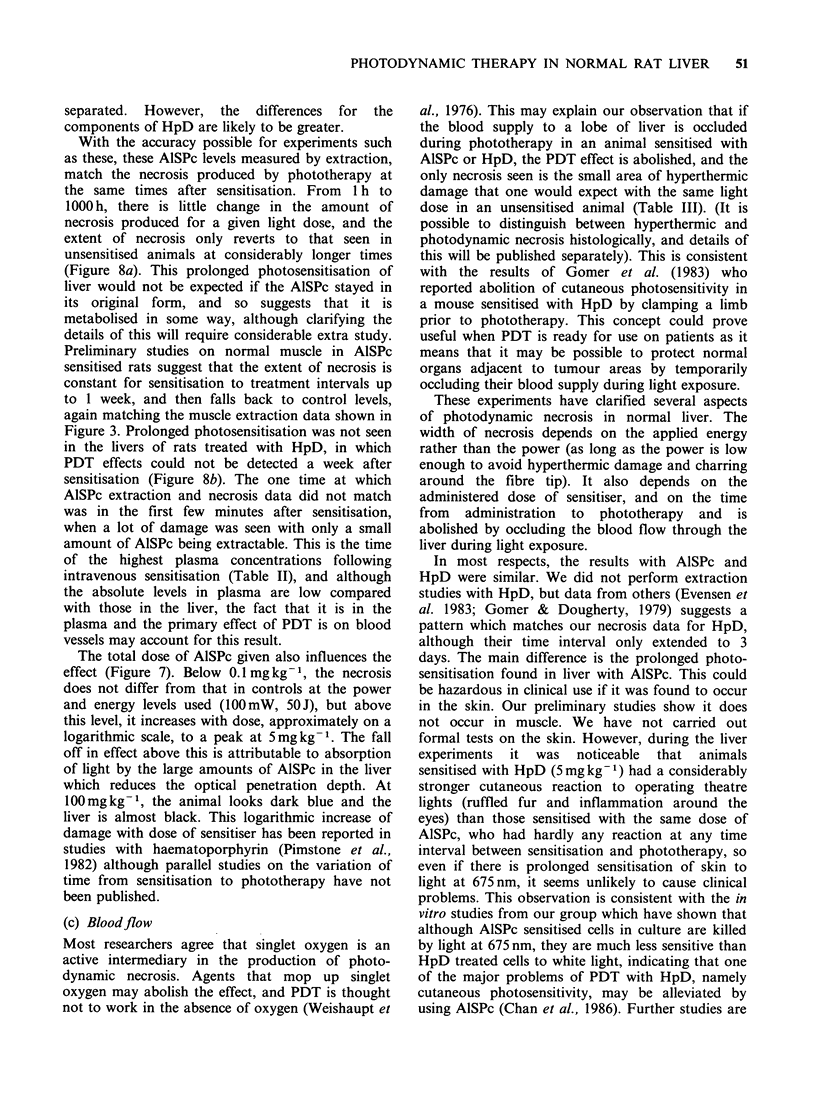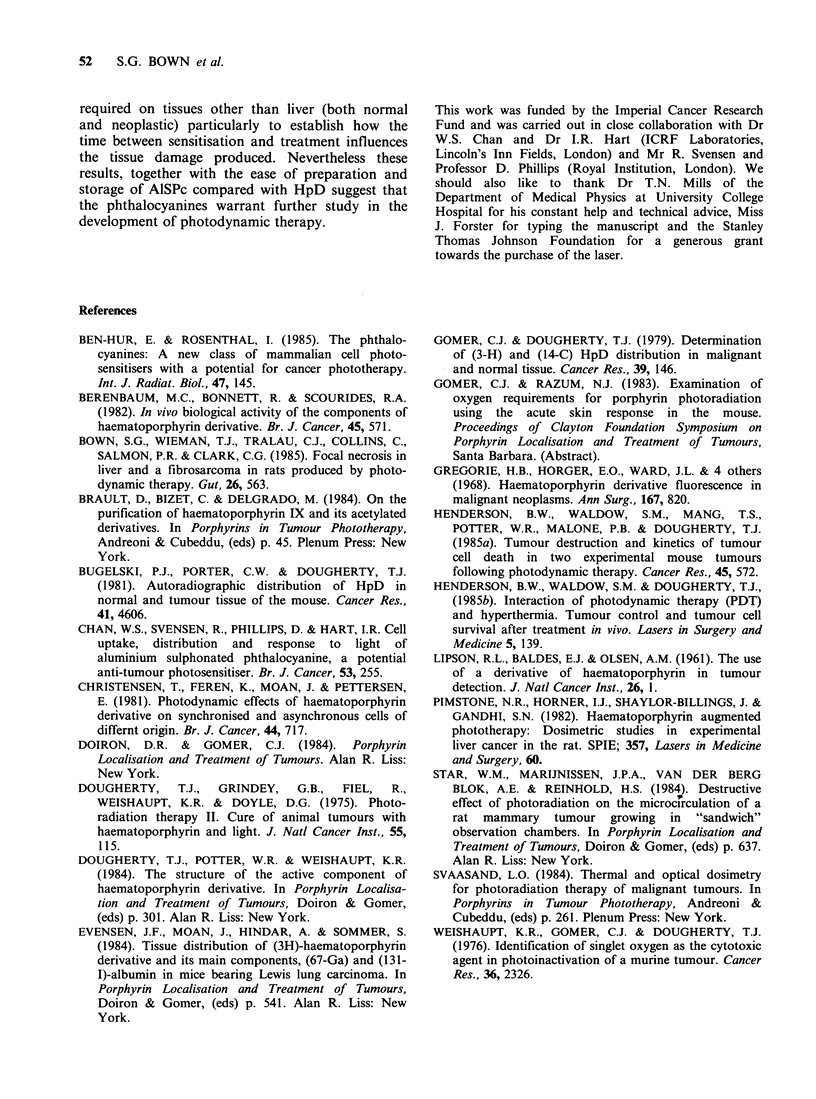# Photodynamic therapy with porphyrin and phthalocyanine sensitisation: quantitative studies in normal rat liver.

**DOI:** 10.1038/bjc.1986.150

**Published:** 1986-07

**Authors:** S. G. Bown, C. J. Tralau, P. D. Smith, D. Akdemir, T. J. Wieman

## Abstract

**Images:**


*Br. J. Cancer* (1986), **54**, 43–52

### Photodynamic therapy with porphyrin and phthalocyanine sensitisation: Quantitative studies in normal rat liver

S.G. Bown, C.J. Tralau, P.D. Coleridge Smith, D. Akdemir & T.J. Wieman

*Rayne Institute, Faculty of Clinical Sciences, University College, London, UK.*

**Summary** Selective sensitisation of malignant tumours to monochromatic light (photodynamic therapy, PDT) is a promising approach to cancer treatment, but current sensitisers are unsatisfactory and the parameters controlling effects produced in normal and neoplastic tissue are poorly understood. To quantify the effects in a relatively homogeneous organ, we carried out experiments in the livers of normal rats following systemic sensitisation with haematoporphyrin derivative (HpD) and a new sensitiser, a sulphonated aluminium phthalocyanine (AlSPc) using light from an Argon pumped tunable dye laser. Damage from PDT (dominant at 100 mW laser power) could be distinguished from that due to local hyperthermia (dominant at 400 mW). For both sensitisers, the extent of PDT necrosis increased with the applied light energy and was abolished by occluding the hepatic blood flow during therapy. With HpD, the extent of PDT necrosis was maximum with only a few hours between sensitisation and therapy, and was not detectable when this interval was increased to a week. With AlSPc, the extent of necrosis in liver changed little with sensitisation times from 1 h to 1000 h (6 weeks), and declined slowly thereafter, matching the amount of AlSPc measurable by alkali extraction, although prolonged photosensitisation was not seen with AlSPc in muscle. Less cutaneous photosensitivity was seen with AlSPc than with HpD. AlSPc is easier to produce and handle than HpD, has a more appropriate strong absorption peak (at 675 nm) and from these results, warrants further study as a photosensitiser for PDT.

Photodynamic therapy (PDT, previously referred to as photoradiation therapy or PRT) has attracted interest in the last few years as a new technique with the potential for selective local destruction of malignant tumours. It is based on the systemic administration of sensitising drugs which may be retained selectively in tumours relative to the surrounding normal tissue and can be activated by light to produce a local cytotoxic effect. Certain aspects of the biological processes involved have been studied in detail, but there are many aspects of the response of both normal and neoplastic tissue to PDT that must be explored before it will be possible to assess what role it may have in the treatment of human disease (Doiron & Gomer, 1984).

Most work has used haematoporphyrin derivative (HpD) as the sensitising agent as this has been shown to provide selective fluorescence in a wide range of human cancers (Gregorie *et al.*, 1968). *In vitro* studies show that HpD is taken up by both normal and neoplastic cell lines in tissue culture and that cell death (probably due to membrane lysis) can be produced by exposure of sensitised cells to light of a wavelength matched to an absorption peak of HpD (usually 630 nm). Unsensitised cells survive the same light dose. However, there is no major difference in the responses of normal and neoplastic cells (Christensen *et al.*, 1981). Nevertheless, studies on transplanted mammary carcinomas in mice suggest that within the tumours, HpD is retained in the vascular stroma, not in individual malignant cells, and that the primary effect of light is to cause a vascular shutdown, necrosis of tumour cells occurring secondary to this (Bugelski *et al.*, 1981, Star *et al.*, 1984) which may explain the selectivity seen in tumours which is not apparent in tissue culture studies. This hypothesis is supported by experiments in which tumour cells were transplanted to tissue culture immediately after phototherapy and grew normally, whereas those transplanted 12 h later were not viable (Henderson *et al.*, 1985*a*). Studies on the regrowth of small transplanted tumours in mice after PDT show that with certain treatment parameters, tumours can be cured and the animals have a normal lifespan (Dougherty *et al.*, 1975). However, few of these reports have looked at more than a small number of the parameters involved and how varying these influences the biological effect. There are no histological studies to follow the effects through from the time of phototherapy until healing is complete, and no studies of what happens at the junction of normal and neoplastic tissue, although this is the most important region when considering

Correspondence: S.G. Bown
Received 13 November 1985; and in revised form, 5 March 1986.

© The Macmillan Press Ltd., 1986

the treatment of human tumours. Despite the fact that some normal tissues take up much more HpD than tumours, only one study has been reported of PDT effects in normal tissue (liver) and they used haematoporphyrin rather than HpD (Pimstone *et al.*, 1982). It is the purpose of the present study to establish which factors control the extent of necrosis produced by PDT in normal liver and to compare a sulphonated aluminium phthalocyanine, AlSPc (the first of a new group of photosensitisers, the phthalocyanines), with HpD (Figure 1). Liver was chosen as it is the organ that achieves the highest concentration of HpD (Gomer & Dougherty, 1979), it is relatively homogeneous and is sufficiently large and accessible in rats to make quantitative studies of the extent of necrosis possible. Such studies are a prerequisite to assessing the effects in tumours. Detailed histological studies of the nature and healing of the damage produced will be published separately.

Although HpD is currently the most studied photosensitiser for PDT, it is far from ideal. It is a poorly defined and probably variable mixture of porphyrins and there is no general agreement on what is the active component (Berenbaum *et al.*, 1982, Dougherty *et al.*, 1984). Tumour selectivity is relatively weak (Gomer & Dougherty, 1979). Also to get adequate light penetration of tissue to treat any lesion greater than 2–3 mm in depth, it is necessary to use a wavelength at the red end of the visible spectrum, and HpD has only a weak absorption peak in this region, at 630 nm. It was because of these problems that we decided to explore the phthalocyanines. AlSPc was chosen as it is relatively easy to synthesise, is chemically stable, is readily soluble in water producing essentially monomeric species and has a strong absorption peak (Q band) in the red part of the spectrum at 675 nm (Figure 2). It has a good fluorescence quantum yield (0.58) which is required if it is to be of value for localisation of small tumours by fluorescence and also a good triplet quantum yield with a long lived triplet state ($510 \pm 50\,\mu$sec at pH 7.4) capable of undergoing bimolecular quenching to produce a reactive species such as singlet oxygen (McCubbin, 1985). Singlet oxygen is thought to be the active intermediary in the cytotoxic effect of PDT (Weishaupt *et al.*, 1976). AlSPc has been reported to be an effective photosensitiser *in vitro* (Ben-Hur *et al.*, 1985) and our own preliminary studies have shown that it has similar distribution in normal and tumour tissue to HpD and can be used as a sensitiser to produce necrosis in normal liver and in a transplanted fibrosarcoma in rats (Bown *et al.*, 1985).

This paper looks at the importance of the dose of the sensitising agent, the time from sensitisation to phototherapy, the power and exposure time of the light source and the circulation through the liver during therapy.

**Figure 1** Basic structure of AlSPc (trisulphonated form is shown) and haematoporphyrin.

### Materials and methods

### (a) *Photosensitisers*

Aluminium Chloro Sulphonated Phthalocyanine (AlSPc), was obtained from Ciba-Geigy and used as received. Sulphonation had been achieved by the action of fuming sulphuric acid on the metallo-phthalocyanine which resulted in an average of three acid groups per molecule (McCubbin, 1985). The AlSPc was dissolved in 0.9% saline for i.v. administration. The solid and solution were kept in the dark, but no other special precautions were taken, and both seem to be chemically stable. Haematoporphyrin obtained in its impure state from Sigma was purified as described by Brault *et al.* (1984) and then treated with acetic acid and sulphuric acid as outlined by Lipson *et al.* (1961). Once made the compound was dried and stored as solid below 0°C in the dark. Solid haematoporphyrin derivative (1 g) was dissolved in 50 ml of 0.1 M sodium hydroxide. After 1 h of stirring the solution was neutralised to pH 7.1 with 0.1 M HCl and adjusted to a total volume of 200 ml with 0.9% NaCl solution giving a solution of concentration 5 mg ml$^{-1}$. This solution was kept in the dark below 0°C (Gomer & Dougherty, 1979).

Experiments were performed on normal Wistar rats (180–250 g). Animals were sensitised by tail vein injections of AlSPc (total dose 0.1 to 100 mg kg$^{-1}$), the concentration of the injected solution being adjusted so the volume of fluid injected was between 0.2 and 1 ml. HpD was only used at 5 mg kg$^{-1}$, prepared as above. All injections and subsequent procedures were carried out under general anaesthetic from intramuscular Hypnorm (fentanyl and fluanisone). This also provided good postoperative analgesia after surgical procedures. Control animals received no sensitising injection.

(b) *Pharmacodynamics of AlSPc*

The concentration of AlSPc in liver, muscle and plasma was measured over various periods of time from a few minutes to several weeks after sensitisation with 5 mg kg$^{-1}$ AlSPc. It was also measured in liver after the administration of various doses (0.2–100 mg kg$^{-1}$) at 3 h from sensitisation to extraction. Rats were killed by cervical dislocation and 0.5 g of liver and of muscle was removed. Each specimen was homogenised in 7 ml of 0.1 M NaOH for 2 min, and the homogenate centrifuged at 12,000 r.p.m. for 5 min at 4°C. The clear supernatant was removed and the fluorescence measured at 678 nm on a spectrofluorimeter (MP4, Perkin Elmer Ltd.). Excitation was at 610 nm with slit width 5 nm. Unfortunately, to date, it has not been possible to attach a radioactive label to AlSPc, so to assess the completeness of the extraction procedure, experiments were carried out on some specimens to repeat the extraction on the pellets remaining after the first centrifugation, and also to assess fluorescence in the pellets after one and two extractions by measuring this after resuspension in a non polar solvent, a 2:1 mixture of chloroform and methanol. In control studies on liver and muscle removed from unsensitised animals and submitted to the same extraction procedure, no fluorescence could be detected at 678 nm.

The effect of quenching was assessed by comparing the fluorescence excited from known concentrations of AlSPc in 0.1 M NaOH with that from the same standard concentrations when present in the supernatant obtained following extractions from unsensitised tissues. Typically, quenching reduced the fluorescence by about 35% through the range of concentrations found in these studies. However, to eliminate the effects of this, our final results from sensitised tissues were calibrated against a standard curve of known concentrations of AlSPc in the supernatant from normal tissue extractions, and expressed as micrograms of extracted AlSPc g$^{-1}$ tissue.

(c) *Phototherapy*

The light source employed was an argon pumped dye laser (Aurora – Cooper Lasersonics). The dye used was DCM (4-Dicyanomethylene-2-Methyl-6 (p-dimethylaminostyryl)-4 H pyran) dissolved in ethylene glycol and propylene carbonate) which enabled the laser to be tuned to emit light either at 630 nm for HpD or 675 nm for AlSPc. The light was delivered via a 0.2 mm diameter quartz fibre, with a plastic coating. The plastic coating was left on to within 1 mm of the fibre tip to reduce the risk of light being emitted from the sides of the fibre proximal to the tip when used with the fibre inserted into the tissue. The tip was cleaved as often as necessary to ensure a clean, circular light beam was produced, and the power emitted from the end of the fibre checked in a separate power meter prior to each treatment.

Phototherapy was carried out on the liver of sensitised and control animals by performing a laparotomy on the rat and exteriorising one lobe of the liver through the incision by gentle traction. The fibre was inserted into the thickest part of the exposed lobe and adjusted so the tip was approximately midway between the upper and lower surfaces of the lobe. The laser, set to give either 100 or 400 mW at the fibre tip, was switched on for the required exposure time after which the fibre track was marked with a loose fine thread suture loop, the fibre removed and the abdomen closed. The animals were killed by cervical dislocation at times from 1 h to 21 days later, although all quantitative results reported in this paper were taken on rats killed 2–4 days after phototherapy. A few minutes before death, the rats were injected with 1 ml of 0.5% Evans blue solution into a tail vein under general anaesthetic which made later identification of necrotic areas easier. At post mortem, the treated lobes of liver were removed, fixed in formalin and sliced at 1 mm intervals in the plain perpendicular to the fibre track (as indicated by the suture marker). The maximum and minimum width of macroscopic necrosis (pale blue or white as compared with darker grey/blue for viable liver) was measured in each slice, and the highest mean value (average of maximum and minimum) recorded as the damage for that liver. Representative sections were prepared for conventional histological examination.

In separate experiments on both control and sensitised animals, the blood flow through the liver was occluded during light exposure by applying a gentle clamp across the base of the lobe. Adequate occlusion was checked by making a small needle prick in the liver distal to the clamp and ensuring that no steady oozing of blood occurred. Preliminary experiments had shown that adequate occlusion was not possible by clamping the hepatic pedicle (hepatic artery and portal vein), presumably because of blood flow from the hepatic vein.

In representative cases at each power level in sensitised and control animals, the light intensity transmitted through the liver parenchyma to the surface was monitored with a small photodiode placed on the liver surface.

Experiments were performed to assess the width of necrosis produced in the liver by varying the following parameters:

(i) Laser power (100 or 400 mW) and exposure time (10 to 2000 sec) and nature of sensitiser (AlSPc or HpD) for one sensitiser dose ($5 mg kg^{-1}$) and one time from sensitisation to treatment (3 h) (wavelength 675 nm for AlSPc and 630 nm for HpD).

(ii) Dose of sensitiser (AlSPc only, 0.1 to $100 mg kg^{-1}$) at one laser power (100 mW), exposure time (500 sec) and time from sensitisation to treatment (3 h).

(iii) Time from sensitisation to treatment (a few minutes to 30 weeks) at one laser power (100 mw), exposure time (500 sec) and dose of sensitiser ($5 mg kg^{-1}$) for both AlSPc and HpD.

(iv) Blood flow through the treated lobe during phototherapy (normal or total occlusion only) at one laser power (100 mW), exposure time (500 sec), dose of sensitiser ($5 mg kg^{-1}$, AlSPc and HpD) and time from sensitisation to treatment (3 h).

### Results

The basic structure of AlSPc is shown in Figure 1. The sensitiser we used is a mixture of the mono-, di-, tri- and tetrasulphonated forms, although it has been estimated that there is an average of three sulphonic acid groups per molecule (McCubbin, 1985). In addition, a range of isomers would have been present. However, each batch we used had the same absorption and fluorescence spectra, which did not change with storage, and similar constant photosensitising properties. This degree of reproducibility has proved extremely difficult to achieve with HpD. There is considerable discussion about the precise nature of the active component of HpD, and it may contain more than one porphyrin ring, but the basic structure of haematoporphyrin is also shown in Figure 1 for comparison. The absorption

**Figure 2** Absorbance spectrum of AlSPc (—) and HpD (---). Each was measured at a concentration of $5 \mu g ml^{-1}$ in undiluted foetal calf serum. The peaks used in this study were 675 nm for AlSPc and 630 nm for HpD.

spectra of the compounds used in our experiments are shown in Figure 2.

The quantity of AlSPc extracted from liver and muscle as a function of the time from injection and the administered dose (liver only) are shown in Figure 3. The results following a second extraction and also measurements of fluorescence in the resuspended pellet after 1 and 2 extractions are shown in Table I. These show that roughly 3 times as much AlSPc is removed by the first extraction as by the second, and that after the second, there is very little fluorescence left in the pellet, so although we do not have an absolute measure of AlSPc levels in tissue, it does appear that we have been able to extract the vast majority of it. The results for plasma are shown in Table II.

**Table I** Fluorescence (in arbitrary units) following NaOH extractions of liver 3 h after intravenous administration of $5 mg kg^{-1}$ AlSPc

| | *After 1 extraction* | *After 2 extractions* |
|---|---|---|
| Supernatant | 23.1 ± 1.0 | 7.5 ± 2.0 |
| Pellet after resuspension in chloroform/methanol | 6.6 ± 2.1 | 1.5 ± 0.7 |

**Table II** Plasma levels of AlSPc measured by alkali extraction following sensitisation with $5 mg kg^{-1}$

| | | | | | |
|---|---|---|---|---|---|
| Time after sensitisation (h) | 0.1 | 1 | 3 | 24 | 48 |
| Concentration of extractable AlSPc ($\mu g ml^{-1}$) | 2.7 | 1.2 | 0.5 | 0 | 0 |

**Figure 3** (*a*) Concentration of extractable AlSPc in normal rat liver (○) and muscle (●) following intravenous administration of $5\,mg\,kg^{-1}$; (*b*) Concentration of extractable AlSPc as a function of dose, 3 h after administration. Each point represents the mean of measurements from 3 livers.

**Figure 4** Section of treated liver following intravenous administration of Evans blue 10 min before death and subsequent fixation in formalin. The area of PDT necrosis (N) is clearly distinguishable from normal areas of liver (L). Magnification ×8.

The typical appearance of treated liver after fixing and sectioning is shown in Figure 4. Detailed histological studies (to be reported in full separately) showed that for given treatment parameters, the maximum extent of tissue damage was visible 24 h after treatment, and this was evident up to 7 days. At longer times, healing occurred with reduction in the lesion size. All measurements of lesion size reported in this paper were made on rats killed 2 to 4 days after treatment.

A direct comparison was made between AlSPc and HpD using each at a dose of $5\,mg\,kg^{-1}$, excited by light of 100 mW power, at the wavelength matched to the maximum absorption of each in the red part of the spectrum (675 nm for AlSPc, 630 nm for HpD). The results for the mean width of necrosis produced against the applied energy (=power × time) are shown in Figure 5 for rats sensitised with AlSPc and HpD, and for unsensitised control animals. At 400 mW, only AlSPc was studied, and the results are shown in Figure 6.

Light intensity measurements made at the surface of the liver during treatment showed that there was no reduction in light transmission during therapy at either wavelength for any of the treatments carried out at 100 mW, but that for all treatments at 400 mW, the intensity dropped to $<10\%$ of the initial value within one minute of starting the light exposure, mainly due to charring on the end of the fibre.

Figure 7 shows the variation in necrosis with the dose of AlSPc and Figure 8 the variation with the time between sensitisation and phototherapy for both AlSPc and HpD. The results of clamping the lobe of the liver during light exposure are shown in Table III.

**Figure 5** Mean diameter of laser induced necrosis in normal liver as a function of the applied energy for exposure at 100 mW, 3 h after sensitisation. Each point with standard deviations represents the results from at least 3 animals. (*a*) AlSPc, 5 mg $kg^{-1}$ (●) and controls (△) treated at 675 nm; (*b*) HpD 5 mg $kg^{-1}$ (●) and controls (△) treated at 630 nm.

**Figure 6** Laser induced necrosis in normal liver as a function of the applied energy for exposure at 400 mW, at 675 nm, 3 h after sensitisation with AlSPc 5 mg $kg^{-1}$ (●) and controls (△).

**Figure 7** Mean diameter of laser induced necrosis in normal liver (100 mW for 500 sec at 675 nm 3 h after sensitisation) as a function of administered dose of AlSPc (○). Control value in unsensitised animals is also shown (▲). Each point represents the results from 3 animals with standard deviations.

**Table III** Effect of occluding the blood supply to the liver during phototherapy. All lesions received 100 mW for 500 sec (50 J) at 3 h after sensitisation

| | *Mean diameter of necrosis (nm)* Clamp (no. of animals) | No Clamp (no. of animals) |
|---|---|---|
| Control (630 nm) | 0.5 ±0 (3) | 1.5 (1) |
| HpD (630 nm) | 0.5 ±0.5 (3) | 3.45±1.4 (5) |
| Control (675 nm) | 0.33±0.2 (3) | 1.8 ±0.7 (5) |
| AlSPc (675 nm) | 0.25±0.4 (3) | 5.5 ±1.2 (6) |

**Figure 8** Mean diameter of laser induced necrosis in normal liver (100 mW, 500 sec) as a function of the time from sensitisation to light exposure. Each point represents the results from at least 3 animals with standard deviations. (*a*) AlSPc 5 mg kg$^{-1}$. Wavelength 675 nm; (*b*) HpD 5 mg kg$^{-1}$. Wavelength 630 nm.

### Discussion

The purpose of this paper is twofold. To establish which factors influence the extent of photodynamic necrosis in normal liver and to compare the previously best studied photosensitiser, HpD, with the newer phthalocyanine (AlSPc). Factors controlling necrosis can be divided into those related to (a) the light dose, (b) the sensitising agent and (c) the blood flow during therapy.

### (a) *Light dose*

When light is delivered to any medium, it can be reflected, transmitted, scattered or absorbed. Only absorbed energy can produce biological effects. The mechanism of PDT is thought to be activation of the sensitiser by absorbed light, conversion of ground state triplet oxygen to the active singlet form by the activated sensitiser and then cell damage (probably by membrane lysis) by the singlet oxygen (Weishaupt *et al.*, 1976). In theory, the damage depends on the total energy delivered rather than the rate at which it is delivered (Svaasand, 1984). The wavelength of light used is chosen to match an absorption peak of the sensitiser and to minimise the absorption by the tissue itself. However, the latter cannot be eliminated entirely, and energy absorbed by the tissue is converted to heat. Whether or not this is sufficient to cause hyperthermic damage depends on the rate at which the energy is delivered (i.e., the power) and the position of the tip of the fibre delivering the light in relation to the tissue being treated.

Our first experiments were designed to distinguish between photodynamic and hyperthermic damage. At 100 mW at both 630 and 675 nm, in the absence of a sensitiser, the diameter of necrosis around the fibre tip (which in this case must be due to hyperthermia) never exceeded 2 mm with energies up to the maximum used (200 J). Thus greater damage produced with similar light doses in the presence of AlSPc or HpD can be attributed to photodynamic effects. This is shown in Figure 5.

On the theoretical grounds, in the region where there is no hyperthermia effect, the total energy fluence of light in the tissue, $W$, at a distance $x$ from the fibre tip is given by the expression

$$W = AW_0 \frac{d}{x} \exp\left(-\left(\frac{x-d}{d}\right)\right) \quad \text{(Svaasand, 1984)}$$

where $W_0$ is the total energy leaving the fibre, $d$ is the optical penetration depth of the liver, which depends on both the absorption and scattering coefficients, and $A$ is a constant which depends on the optics and geometry of the fibre tip.

The tissue damage depends on the total energy absorbed, so if $r$ is the radius of the necrotic zone, this will be related to the threshold fluence rquired to produce necrosis, $W_T$, by the expression

$$W_T = A W_0 \frac{d}{r} \exp\left(-\left(\frac{r-d}{d}\right)\right)$$

Thus $$W_0 = \frac{W_T}{A}\left(\frac{r}{d}\right)\exp\left(\frac{r-d}{d}\right) \quad (1)$$

In Figure 5, the diameter of necrosis produced ($=2r$) is plotted, so values of $r$ range from 0.5 to 4.3 mm. From Svaasand (1984), $d$ is of the order of 1.5–2 mm in liver, and to a first approximation, when $r$ and $d$ have similar values,

$$\ln\left(\frac{r}{d}\right) \approx \frac{r}{d} - 1 \quad (2)$$

From (1),

$$\ln W_0 = \ln\left(\frac{r}{d}\right) + \ln\left(\frac{W_T}{A}\right) + \left(\frac{r-d}{d}\right)$$

Substituting from (2),

$$2r = d \ln W_0 - d \ln \frac{W_T}{A} + 2$$

Thus, in the range of values found in our results, Figures 5(a) and (b) should be straight lines with gradient $d$.

Using a least squares fit to these data, the optical penetration depth, $d$, for AlSPc sensitised liver at 675 nm is 1.8 mm, and for HpD sensitised liver at 630 nm is 1.2 mm. The latter figure is consistent with that reported by Svaasand (1984) for studies at 630 nm and it is reassuring that his figure, measured by direct recording of light transmitted through liver is consistent with ours which is based on the biological effect of the light. These results also agree with those of Pimstone *et al.* (1982) who, using an external light source showed that the depth of necrosis increased with the natural logarithm of the applied energy. In addition, they showed that under their experimental conditions the necrosis was independent of the power of the light source (as would be expected as long as hyperthermic conditions were not obtained (Svaasand, 1984)) although they did not report any control studies to exclude hyperthermic effects. At the sensitiser concentrations used in this aspect of our study, the presence of the AlSPc or HpD is not likely to alter the optical properties of the liver significantly so the increased penetration at 675 nm is purely a wavelength effect, and supports the suggestion that larger volumes of tissue can be treated from one treatment site at this wavelength than at 630 nm for given light doses. The scatter seen in our measurements may be due to biological variations between animals, movement of the rat liver with respiration, which could cause the fibre tip to move during exposure or, if the mechanism of PDT is indeed occlusion of the vasculature, the damage could depend critically on the location of the fibre tip in relation to blood vessels, which is difficult to ascertain.

In contrast to the effects at 100 mW when the intensity of light transmission did not fall during exposure, when the laser power was increased to 400 mW, the transmission through the liver dropped rapidly during therapy, typically falling to 10% of the original value within 1 min (with or without sensitiser) due to charring occurring on the fibre tip. With such a rapid and dramatic loss of light transmission in the red part of the spectrum (which is required to activate the sensitiser), further energy transmission through the liver can only be thermal, so it is not surprising in Figure 6 that for exposures longer than 1 min or so ($=24$ J at 400 mW) there is little difference between the necrosis produced with and without the sensitiser. The number of experiments carried out at 400 mW with exposure times $<1$ min (i.e. when there is still reasonable transmission of red light) was small, due to technical difficulties but in those with AlSPc the damage is greater than would be expected for the same energies at 100 mW in similarly sensitised animals. This could be due to synergy between hyperthermia and PDT, as has been reported for HpD (Henderson *et al.*, 1985*b*). Higher powers could be used without the risk of charring and with less risk of producing hyperthermia effects either by holding the fibre tip above the surface of the treated organ (external exposure) or by using a different fibre tip in which the light is emitted from a larger surface area than just the bare tip (diffuser fibres).

(b) *Sensitiser*

In the experiments described in this paper, rats were sensitised with a single bolus dose of AlSPc, given into a tail vein. The sodium hydroxide extraction studies (Figure 3) show that after 5 mg kg$^{-1}$, the concentration in liver is maximum after $\sim$3 h, but does not start falling significantly until $\sim$1000 h (6 weeks) after sensitisation and only becomes undetectable after several thousand hours. For these pharmacokinetic studies to be valid, one must assume that the components with different numbers of sulphonic acid groups are handled similarly by the liver over this time period, and this is difficult to prove until the components can be separated. However, the differences for the components of HpD are likely to be greater.

With the accuracy possible for experiments such as these, these AlSPc levels measured by extraction, match the necrosis produced by phototherapy at the same times after sensitisation. From 1 h to 1000 h, there is little change in the amount of necrosis produced for a given light dose, and the extent of necrosis only reverts to that seen in unsensitised animals at considerably longer times (Figure 8*a*). This prolonged photosensitisation of liver would not be expected if the AlSPc stayed in its original form, and so suggests that it is metabolised in some way, although clarifying the details of this will require considerable extra study. Preliminary studies on normal muscle in AlSPc sensitised rats suggest that the extent of necrosis is constant for sensitisation to treatment intervals up to 1 week, and then falls back to control levels, again matching the muscle extraction data shown in Figure 3. Prolonged photosensitisation was not seen in the livers of rats treated with HpD, in which PDT effects could not be detected a week after sensitisation (Figure 8*b*). The one time at which AlSPc extraction and necrosis data did not match was in the first few minutes after sensitisation, when a lot of damage was seen with only a small amount of AlSPc being extractable. This is the time of the highest plasma concentrations following intravenous sensitisation (Table II), and although the absolute levels in plasma are low compared with those in the liver, the fact that it is in the plasma and the primary effect of PDT is on blood vessels may account for this result.

The total dose of AlSPc given also influences the effect (Figure 7). Below 0.1 mg kg$^{-1}$, the necrosis does not differ from that in controls at the power and energy levels used (100 mW, 50 J), but above this level, it increases with dose, approximately on a logarithmic scale, to a peak at 5 mg kg$^{-1}$. The fall off in effect above this is attributable to absorption of light by the large amounts of AlSPc in the liver which reduces the optical penetration depth. At 100 mg kg$^{-1}$, the animal looks dark blue and the liver is almost black. This logarithmic increase of damage with dose of sensitiser has been reported in studies with haematoporphyrin (Pimstone *et al.*, 1982) although parallel studies on the variation of time from sensitisation to phototherapy have not been published.

(c) *Blood flow*

Most researchers agree that singlet oxygen is an active intermediary in the production of photodynamic necrosis. Agents that mop up singlet oxygen may abolish the effect, and PDT is thought not to work in the absence of oxygen (Weishaupt *et al.*, 1976). This may explain our observation that if the blood supply to a lobe of liver is occluded during phototherapy in an animal sensitised with AlSPc or HpD, the PDT effect is abolished, and the only necrosis seen is the small area of hyperthermic damage that one would expect with the same light dose in an unsensitised animal (Table III). (It is possible to distinguish between hyperthermic and photodynamic necrosis histologically, and details of this will be published separately). This is consistent with the results of Gomer *et al.* (1983) who reported abolition of cutaneous photosensitivity in a mouse sensitised with HpD by clamping a limb prior to phototherapy. This concept could prove useful when PDT is ready for use on patients as it means that it may be possible to protect normal organs adjacent to tumour areas by temporarily occluding their blood supply during light exposure.

These experiments have clarified several aspects of photodynamic necrosis in normal liver. The width of necrosis depends on the applied energy rather than the power (as long as the power is low enough to avoid hyperthermic damage and charring around the fibre tip). It also depends on the administered dose of sensitiser, and on the time from administration to phototherapy and is abolished by occluding the blood flow through the liver during light exposure.

In most respects, the results with AlSPc and HpD were similar. We did not perform extraction studies with HpD, but data from others (Evensen *et al.* 1983; Gomer & Dougherty, 1979) suggests a pattern which matches our necrosis data for HpD, although their time interval only extended to 3 days. The main difference is the prolonged photosensitisation found in liver with AlSPc. This could be hazardous in clinical use if it was found to occur in the skin. Our preliminary studies show it does not occur in muscle. We have not carried out formal tests on the skin. However, during the liver experiments it was noticeable that animals sensitised with HpD (5 mg kg$^{-1}$) had a considerably stronger cutaneous reaction to operating theatre lights (ruffled fur and inflammation around the eyes) than those sensitised with the same dose of AlSPc, who had hardly any reaction at any time interval between sensitisation and phototherapy, so even if there is prolonged sensitisation of skin to light at 675 nm, it seems unlikely to cause clinical problems. This observation is consistent with the *in vitro* studies from our group which have shown that although AlSPc sensitised cells in culture are killed by light at 675 nm, they are much less sensitive than HpD treated cells to white light, indicating that one of the major problems of PDT with HpD, namely cutaneous photosensitivity, may be alleviated by using AlSPc (Chan *et al.*, 1986). Further studies are required on tissues other than liver (both normal and neoplastic) particularly to establish how the time between sensitisation and treatment influences the tissue damage produced. Nevertheless these results, together with the ease of preparation and storage of AlSPc compared with HpD suggest that the phthalocyanines warrant further study in the development of photodynamic therapy.

This work was funded by the Imperial Cancer Research Fund and was carried out in close collaboration with Dr W.S. Chan and Dr I.R. Hart (ICRF Laboratories, Lincoln's Inn Fields, London) and Mr R. Svensen and Professor D. Phillips (Royal Institution, London). We should also like to thank Dr T.N. Mills of the Department of Medical Physics at University College Hospital for his constant help and technical advice, Miss J. Forster for typing the manuscript and the Stanley Thomas Johnson Foundation for a generous grant towards the purchase of the laser.

**References**

BEN-HUR, E. & ROSENTHAL, I. (1985). The phthalocyanines: A new class of mammalian cell photosensitisers with a potential for cancer phototherapy. *Int. J. Radiat. Biol.*, **47**, 145.

BERENBAUM, M.C., BONNETT, R. & SCOURIDES, R.A. (1982). *In vivo* biological activity of the components of haematoporphyrin derivative. *Br. J. Cancer*, **45**, 571.

BOWN, S.G., WIEMAN, T.J., TRALAU, C.J., COLLINS, C., SALMON, P.R. & CLARK, C.G. (1985). Focal necrosis in liver and a fibrosarcoma in rats produced by photodynamic therapy. *Gut*, **26**, 563.

BRAULT, D., BIZET, C. & DELGRADO, M. (1984). On the purification of haematoporphyrin IX and its acetylated derivatives. In *Porphyrins in Tumour Phototherapy*, Andreoni & Cubeddu, (eds) p. 45. Plenum Press: New York.

BUGELSKI, P.J., PORTER, C.W. & DOUGHERTY, T.J. (1981). Autoradiographic distribution of HpD in normal and tumour tissue of the mouse. *Cancer Res.*, **41**, 4606.

CHAN, W.S., SVENSEN, R., PHILLIPS, D. & HART, I.R. Cell uptake, distribution and response to light of aluminium sulphonated phthalocyanine, a potential anti-tumour photosensitiser. *Br. J. Cancer*, **53**, 255.

CHRISTENSEN, T., FEREN, K., MOAN, J. & PETTERSEN, E. (1981). Photodynamic effects of haematoporphyrin derivative on synchronised and asynchronous cells of differnt origin. *Br. J. Cancer*, **44**, 717.

DOIRON, D.R. & GOMER, C.J. (1984). *Porphyrin Localisation and Treatment of Tumours*. Alan R. Liss: New York.

DOUGHERTY, T.J., GRINDEY, G.B., FIEL, R., WEISHAUPT, K.R. & DOYLE, D.G. (1975). Photoradiation therapy II. Cure of animal tumours with haematoporphyrin and light. *J. Natl Cancer Inst.*, **55**, 115.

DOUGHERTY, T.J., POTTER, W.R. & WEISHAUPT, K.R. (1984). The structure of the active component of haematoporphyrin derivative. In *Porphyrin Localisation and Treatment of Tumours*, Doiron & Gomer, (eds) p. 301. Alan R. Liss: New York.

EVENSEN, J.F., MOAN, J., HINDAR, A. & SOMMER, S. (1984). Tissue distribution of (3H)-haematoporphyrin derivative and its main components, (67-Ga) and (131-I)-albumin in mice bearing Lewis lung carcinoma. In *Porphyrin Localisation and Treatment of Tumours*, Doiron & Gomer, (eds) p. 541. Alan R. Liss: New York.

GOMER, C.J. & DOUGHERTY, T.J. (1979). Determination of (3-H) and (14-C) HpD distribution in malignant and normal tissue. *Cancer Res.*, **39**, 146.

GOMER, C.J. & RAZUM, N.J. (1983). Examination of oxygen requirements for porphyrin photoradiation using the acute skin response in the mouse. *Proceedings of Clayton Foundation Symposium on Porphyrin Localisation and Treatment of Tumours*, Santa Barbara. (Abstract).

GREGORIE, H.B., HORGER, E.O., WARD, J.L. & 4 others (1968). Haematoporphyrin derivative fluorescence in malignant neoplasms. *Ann Surg.*, **167**, 820.

HENDERSON, B.W., WALDOW, S.M., MANG, T.S., POTTER, W.R., MALONE, P.B. & DOUGHERTY, T.J. (1985*a*). Tumour destruction and kinetics of tumour cell death in two experimental mouse tumours following photodynamic therapy. *Cancer Res.*, **45**, 572.

HENDERSON, B.W., WALDOW, S.M. & DOUGHERTY, T.J., (1985*b*). Interaction of photodynamic therapy (PDT) and hyperthermia. Tumour control and tumour cell survival after treatment *in vivo*. *Lasers in Surgery and Medicine* **5**, 139.

LIPSON, R.L., BALDES, E.J. & OLSEN, A.M. (1961). The use of a derivative of haematoporphyrin in tumour detection. *J. Natl Cancer Inst.*, **26**, 1.

PIMSTONE, N.R., HORNER, I.J., SHAYLOR-BILLINGS, J. & GANDHI, S.N. (1982). Haematoporphyrin augmented phototherapy: Dosimetric studies in experimental liver cancer in the rat. SPIE; **357**, *Lasers in Medicine and Surgery*, **60**.

STAR, W.M., MARIJNISSEN, J.P.A., VAN DER BERG BLOK, A.E. & REINHOLD, H.S. (1984). Destructive effect of photoradiation on the microcirculation of a rat mammary tumour growing in "sandwich" observation chambers. In *Porphyrin Localisation and Treatment of Tumours*, Doiron & Gomer, (eds) p. 637. Alan R. Liss: New York.

SVAASAND, L.O. (1984). Thermal and optical dosimetry for photoradiation therapy of malignant tumours. In *Porphyrins in Tumour Phototherapy*, Andreoni & Cubeddu, (eds) p. 261. Plenum Press: New York.

WEISHAUPT, K.R., GOMER, C.J. & DOUGHERTY, T.J. (1976). Identification of singlet oxygen as the cytotoxic agent in photoinactivation of a murine tumour. *Cancer Res.*, **36**, 2326.